# Design and processor in the loop implementation of an improved control for IM driven solar PV fed water pumping system

**DOI:** 10.1038/s41598-022-08252-7

**Published:** 2022-03-18

**Authors:** Mustapha Errouha, Quentin Combe, Saad Motahhir, S. S. Askar, Mohamed Abouhawwash

**Affiliations:** 1 Plasma and Conversion of Energy Laboratory, ENSEEIHT, University of Toulouse, Toulouse, France; 2 LEMTA, University of Lorraine, Vandœuvre-lès-Nancy, France; 3 ENSA, SMBA University, Fez, Morocco; 4 Department of Statistics and Operations Research, College of Science, King Saud University, Riyadh, 11451 Saudi Arabia; 5 Department of Mathematics, Faculty of Science, Mansoura University, Mansoura, 35516 Egypt; 6 Department of Computational Mathematics, Science, and Engineering (CMSE), College of Engineering, Michigan State University, East Lansing, MI 48824 USA

**Keywords:** Energy science and technology, Physics

## Abstract

In recent years, the improvement of photovoltaic water pumping system (PVWPS) efficiency takes the considerable interest of researchers due to its operating based on cleaner electrical energy production. In this paper, a new approach based on fuzzy logic controller incorporating loss minimization technique applied to the induction machine (IM) is developed for PVWPS applications. The proposed control selects the optimal flux magnitude by minimization of the IM losses. Moreover, Variable step size perturb and observe method is introduced. The suitability of the proposed control is approved by reducing the absorbed current; therefore, the motor losses are minimized and the efficiency is improved. The proposed control strategy is compared with the method without losses minimization. The comparison results illustrate the effectiveness of the proposed method based on losses minimization regarding the electrical speed, absorbed current, flow water and developed flux. A processor-in-the-loop (PIL) test is effectuated as an experimental test of the proposed method. It consists in implementing the generated C code on the STM32F4 discovery board. The obtained results from the embedded board are similar to numerical simulation results.

## Introduction

Renewable energy sources especially solar PV technology can be a cleaner alternative solution to fossils fuels for water pumping systems^1,2^. PV water pumping system is gained a lot of attention in remote areas where electricity is not available^3,4^.

Various kinds of engines are utilized with PV pumping applications. The primitive stage of PVWPS is based on DC motor. These motors are easy to control and implement but they need regular maintenance due to commentators and brushes^5^. To overcome this disadvantage, Brushless permanent magnet motors are introduced which are characterized by the absence of brushes, high efficiency and reliability^6^. PVWPS based on IM illustrates better performance compared to other motors because this type of motor is reliable, low cost and maintenance-free and gives more possibilities for control strategies^7^. Indirect Field oriented control (IFOC) technique and direct torque control (DTC) method are often employed^8^.

IFOC was developed by Blaschke and Hasse to allow varying IM speed over a wide range^9,10^. The stator currents are separated into two components, one generates the flux and the other produces the torque by utilizing transformation to the d–q coordinate system. This allows independent control of the flux and torque during both the steady state and dynamic conditions. The axis (d) is aligned with the rotor flux space vector which involves that the q-axis component of the rotor flux space vector is always zero. FOC gives a good and faster response^11,12^, however, this method is complex and affected by the parameter variations^13^. To surmount these drawbacks, DTC was introduced by Takashi and Noguchi^14^, this command presents high dynamic performance, and it is robust and less sensitive to parameter variations. In DTC, the control of the electromagnetic torque and the stator flux is made using subtracting the stator flux and torque from the corresponding estimated values. The result is introduced to hysteresis comparators to generate the appropriate voltage vectors to control simultaneously the stator flux and the torque.

The major inconvenience of this control strategy is high ripples in torque and flux due to the use of the hysteresis regulators for stator flux and electromagnetic torque regulation^15,42^. The multilevel converters are used for minimizing the ripples but the efficiency is reduced due to the number of power switches^16^. Several authors have used Space Vector Modulation (SWM)^17^, Sliding mode control (SMC)^18^, this technique is robust but the undesirable chattering effect is appeared^19^. Many researchers used the artificial intelligence techniques to improve the controller performances, among them, (1) neural network, this control strategy requires a high-speed processor for implementation^20^, (2) genetic algorithm^21^.

The fuzzy control is robust, suitable for the nonlinear control strategy and it does not demand the knowledge of the exact model. It consists in using the fuzzy logic block instead of the hysteresis controllers and the switching selection table to reduce the flux and torque ripples. It is worth indicating that DTC based on FLC offers better performance^22^, but it isn't sufficient to maximize the efficiency of the engine, therefore an optimization technique is needed with the control loop.

In most of the previous studies, the authors choose a constant flux as reference flux^23–26^, but this choice of reference doesn’t represent the optimum operating.

The high-performance efficient motor drives require fast and accurate speed response. On the other hand, the control can be non-optimal for some operations and hence, the efficiency of the drive system cannot be optimized. The use of variable flux reference during the operating of the system can achieve better performance.

Many authors proposed the search controller (SC) that minimizes the losses for the improvement of the efficiency of the engine at different load conditions such as in^27^. This technique consists in measuring and minimizing the input power by iterating the reference of d-axis current or the stator flux reference. However, this approach introduces torque ripples due to the oscillations present in the air gap flux and the implementation of this method is time consuming and computational resource intensive. Particle swarm optimization is also used to improve efficiency^28^, but this technique can be trapped into a local minimum which leads to improperly chosen control parameters^29^.

In this paper, a technique associated to FDTC to select the optimal flux by reducing the motor losses is proposed. This combination ensures the functioning using the optimal flux level at each operating point, which enhances the efficiency of the proposed PV water pumping system. Hence, it appears to be very convenient for PV water pumping applications.

Besides, a processor in the loop test is conducted as an experimental verification of the proposed method using STM32F4 board. The main advantages of this core are simplicity of implementation, low cost and no necessity to develop a complex program^30^. Moreover, the FT232RL USB‐UART converter board is associated with STM32F4 to ensure an external communication interface in order to establish a virtual serial port on the computer (COM port). This method allows the transmission of data at a high baud rate.

The performance of the PVWPS using the proposed technique is compared with the PV system without losses minimization under different operating conditions. The obtained results show that the proposed PV water pumping system is better in terms of minimization of stator current and copper losses, optimizing flux and pumped water.

The rest of the paper is structured as follows: the modeling of the proposed system is given in “Modeling of PV system” section. In “Control strategies for the studied system” section, FDTC, the proposed control strategies and MPPT technique are detailed. The research results are discussed in “Simulation results” section. In “PIL test using STM32F4 discovery board” section, the processor in the loop test is presented. The conclusions of this paper are presented in “Conclusion” section.

## Modeling of PV system

**Figure 1:**
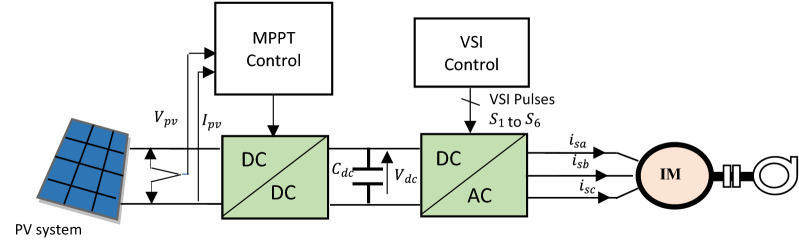
Description of the proposed system.

Figure 1 shows the system configuration for the proposed standalone PV water pumping system. The system is composed of an IM based centrifugal pump, a PV array, two power converters [boost converters and voltage source inverter (VSI)]. In this section, the modeling of the studied PV water pumping system is presented.

### Photovoltaic cell

The single diode model of cell the solar photovoltaic cell is adopted in this work. The characteristic of PV cell is expressed by^31–33^.

1 $$I = I_{ph} {\text{ - I}}_{0} \left( {{\text{exp}}\frac{{q\left( {V + R_{ss} I} \right)}}{{{\text{aKTN}}_{s} }}{ - 1}} \right) - \frac{{\left( {V + IR_{ss} } \right)}}{{R_{{{\text{sh}}}} }}$$

### DC-DC converter

To perform an adaptation, the boost converter is employed. The relation between input and output voltages of the DC-DC converter is given by^34^:

2 $$V_{dc} = \frac{{V_{pv} }}{1 - \alpha }$$

### DC–AC converter

The equations that characterize the behavior of the DC–AC converter are expressed by^35,41^:

3 $$\left[ {\begin{array}{*{20}c} {V_{a} } \\ {V_{b} } \\ {V_{c} } \\ \end{array} } \right] = \frac{{V_{dc} }}{3}\left[ {\begin{array}{*{20}c} 2 & { - 1} & { - 1} \\ { - 1} & 2 & { - 1} \\ { - 1} & { - 1} & 2 \\ \end{array} } \right]\left[ {\begin{array}{*{20}c} {S_{a} } \\ {S_{b} } \\ {S_{c} } \\ \end{array} } \right]$$

### Induction motor

the mathematical model of the IM can be described in the reference frame (α,β) by the following equations^5,40^:

$$\left\{ {\begin{array}{*{20}l} {V_{\alpha s} = R_{s } I_{\alpha s } + \frac{d}{dt}\phi_{\alpha s} \quad (4)} \hfill \\ {V_{\beta s} = R_{s } I_{\beta s } + \frac{d}{dt}\phi_{\beta s} \quad (5)} \hfill \\ \end{array} } \right.$$

$$\left\{ {\begin{array}{*{20}c} {\phi_{\alpha s} = l_{s } I_{\alpha s } + MI_{\alpha s} \quad (6)} \\ {\phi_{\beta s} = l_{s } I_{\beta s } + MI_{\beta s} \quad (7)} \\ \end{array} } \right.$$

$$\left\{ {\begin{array}{*{20}c} {0 = I_{\alpha r } R_{r } + \frac{d}{dt}\phi_{\alpha r} + \phi_{\beta r} w_{m} \quad (8)} \\ {0 = I_{\beta r } R_{r } + \frac{d}{dt}\phi_{\beta r} + \phi_{\alpha r} w_{m} \quad (9) } \\ \end{array} } \right.$$

$$\left\{ {\begin{array}{*{20}c} {\phi_{\alpha r} = I_{\alpha r } l_{r} + MI_{\alpha r} \quad (10)} \\ {\phi_{\beta r} = I_{\beta r } l_{r } + MI_{\beta r} \quad (11)} \\ \end{array} } \right.$$

and the electromagnetic torque developed:

12 $${ } T_{em} = { }\frac{3}{2}{ }P\left( {\phi_{\alpha s} I_{\beta s} { } - { }\phi_{\beta s} I_{\alpha s} { }} \right)$$

where $l_{s }$,$l_{r}$ : Stator and Rotor inductances, M: mutual inductance, $R_{s }$,$I_{s }$: Stator resistance and stator current, $R_{r}$,$I_{r }$: Rotor resistance and rotor current, $\phi_{s}$ , $V_{s}$ : Stator flux and stator voltage, $\phi_{r}$, $V_{r}$ : Rotor flux and rotor voltage.

### Pump

The load torque of the centrifugal pump which is in proportion to the square of the IM speed can be determined by:

13 $$T_{r} = K_{p} \Omega^{2}$$

## Control strategies for the studied system

The control of the proposed water pumping system is divided into three different subsections. First section deals with the MPPT technique. The second part deals with the direct torque control based on fuzzy logic controller to drive the IM. Moreover, the third part describes a technique associated with DTC based on FLC, which allows determining the reference flux.

### MPPT technique

**Figure 2:**
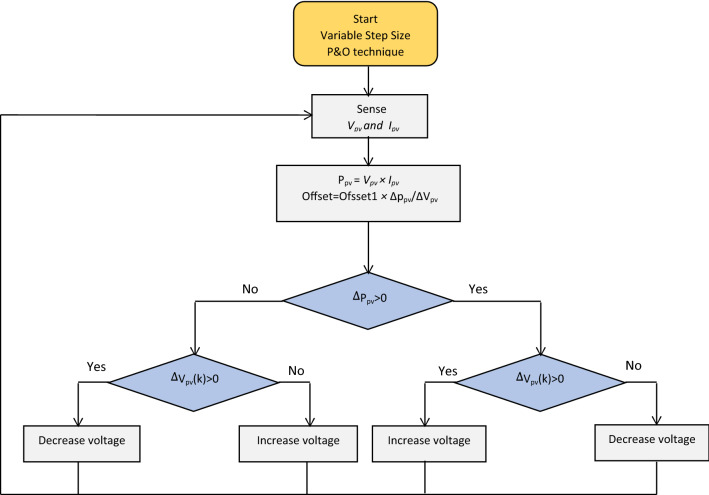
flowchart of Variable step size P&O method.

In this work, Variable Step Size P&O technique is employed for tracking of maximum power point. It’s characterized by fast tracking and low oscillations (Fig. 2)^37–39^.

### DTC based on fuzzy logic controller

The principal idea of the DTC is to directly command the flux and torque of the machine, but the use of the hysteresis regulators for electromagnetic torque and stator flux regulation leads to high torque and flux ripples. Thus, a fuzzy technique is introduced to enhance the DTC method (Fig. 7), The FLC can develop the adequate inverter vector state.

The stator flux components can be expressed by:

14 $$\left\{ {\begin{array}{*{20}c} {\hat{\varphi }_{s\alpha } = \mathop \smallint \limits_{0}^{t} \left( {v_{s\alpha } - R_{s} \cdot i_{s\alpha } } \right) \cdot dt} \\ {\hat{\varphi }_{s\beta } = \mathop \smallint \limits_{0}^{t} \left( {v_{s\beta } - R_{s} \cdot i_{s\beta } } \right) \cdot dt} \\ \end{array} } \right.$$

The estimated electromagnetic torque can be written as:

15 $$\hat{T}_{em} = p \cdot \left( {\hat{\varphi }_{s\alpha } \cdot i_{s\beta } - \hat{\varphi }_{s\beta } \cdot i_{s\alpha } } \right)$$

In addition, the stator flux angle and the amplitude are given by:

16 $$\hat{\varphi }_{s} = \sqrt {\hat{\varphi }_{s\alpha }^{2} + \hat{\varphi }_{s\beta }^{2} }$$

17 $$\theta_{s} = arctg\left( {\frac{{\hat{\varphi }_{s\beta } }}{{\hat{\varphi }_{s\alpha } }}} \right)$$

An FLC is generally composed of four main steps:

#### Fuzzification

During this step, the inputs are converted into fuzzy variables through membership functions (MFs) and linguistic terms.

#### For stator flux error

**Figure 3:**
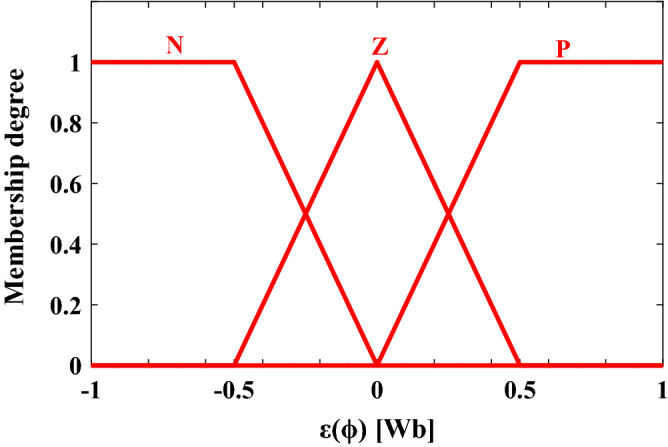
The fuzzy membership functions of ε_φ_.

The three membership functions for the first input (ε_φ_) are negative (N), positive (P) and zero (Z) as shown in Fig. 3.

#### For torque error

**Figure 4:**
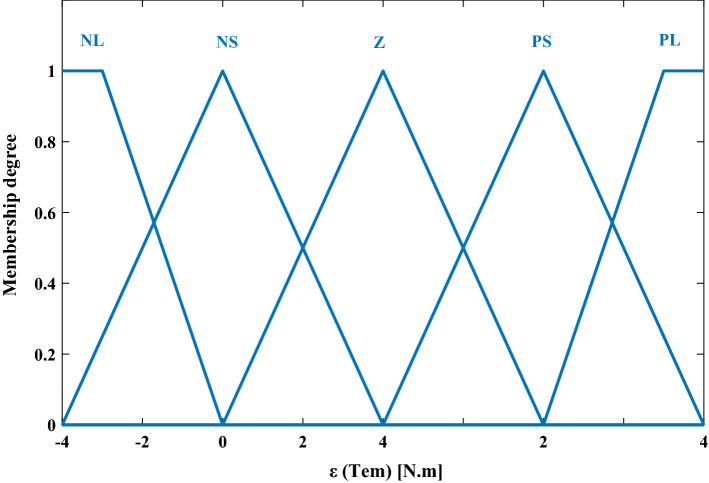
The fuzzy membership functions of $\varepsilon$_Tem_.

The five membership functions for the second input ($\varepsilon$_Tem_) are negative large (NL) negative small (NS) zero (Z) positive small (PS) and positive large (PL) as shown in Fig. 4.

#### For the sector angle

**Figure 5:**
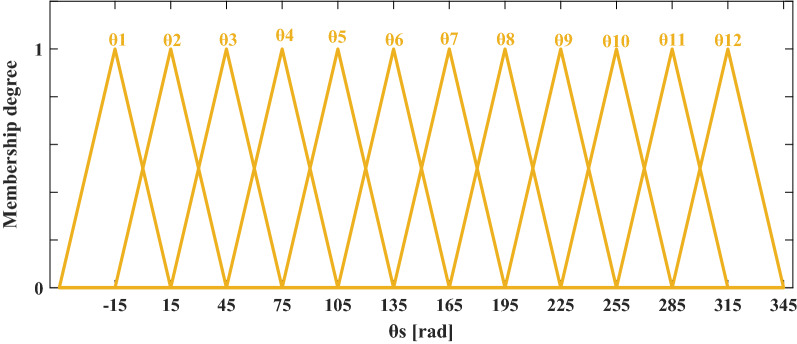
The fuzzy membership functions of θ_s_.

The stator flux trajectory consists of 12 sectors in which the fuzzy sets are represented by isosceles triangular membership functions as shown in Fig. 5.

#### Fuzzy control rules

Table 1 groups 180 fuzzy rules which are determined using membership functions of the inputs to select the suitable switching state.

**Table 1:** Fuzzy switching logic rule base.

ε(φ)	ε(Tem)	${\uptheta }_{ 1}$	${\theta }_{2}$	${\theta }_{3}$	${\uptheta }_{ 4}$	${\theta }_{5}$	${\uptheta }_{ 6}$	${\uptheta }_{ 7}$	${\uptheta }_{ 8}$	${\uptheta }_{ 9}$	${\uptheta }_{ 10}$	${\uptheta }_{ 11}$	${\theta }_{12}$
NL	Z	$V _{6}$	$V _{1}$	$V _{1}$	$V _{2}$	$V_{ 2}$	$V_{ 3}$	$V _{3}$	$V _{4}$	$V _{4}$	$V _{5}$	$V _{5}$	$V _{6}$
PL		$V_{ 2}$	$V_{ 3}$	$V _{3}$	$V_{ 4}$	$V_{ 4}$	$V_{ 5}$	$V _{5}$	$V _{6}$	$V _{6}$	$V_{ 1}$	$V _{1}$	$V _{2}$
Z		$V_{ 7}$	$V_{ 0}$	$V _{7}$	$V_{ 0}$	$V_{ 7}$	$V _{0}$	$V _{7}$	$V _{0}$	$V_{ 7}$	$V _{0}$	$V_{ 7}$	$V_{ 0}$
PS		$V_{ 2}$	$V_{ 3}$	$V _{3}$	$V_{ 4}$	$V _{4}$	$V _{5}$	$V _{5}$	$V _{6}$	$V _{6}$	$V_{ 1}$	$V_{ 1}$	$V _{2}$
NS		$V_{ 7}$	$V _{0}$	$V_{ 7}$	$V _{0}$	$V _{7}$	$V _{0}$	$V_{ 7}$	$V _{0}$	$V_{ 7}$	$V _{0}$	$V_{ 7}$	$V _{0}$
PL	P	$V _{2}$	$V _{3}$	$V _{3}$	$V _{4}$	$V _{4}$	$V _{5}$	$V_{ 5}$	$V_{ 6}$	$V _{6}$	$V _{1}$	$V _{1}$	$V _{2}$
NL		$V _{6}$	$V_{ 1}$	$V_{ 1}$	$V _{2}$	$V_{ 2}$	$V _{3}$	$V _{3}$	$V_{ 4}$	$V _{4}$	$V _{5}$	$V _{5}$	$V _{6}$
Z		$V _{0}$	$V_{ 7}$	$V _{0}$	$V_{ 7}$	$V _{0}$	$V_{ 7}$	$V _{0}$	$V_{ 7}$	$V _{0}$	$V _{7}$	$V_{ 0}$	$V _{7}$
PS		$V _{2}$	$V_{ 2}$	$V_{ 3}$	$V _{3}$	$V _{4}$	$V _{4}$	$V _{5}$	$V_{ 5}$	$V _{6}$	$V _{6}$	$V _{1}$	$V _{1}$
NS		$V _{1}$	$V _{1}$	$V _{2}$	$V _{2}$	$V _{3}$	$V _{3}$	$V _{4}$	$V _{4}$	$V _{5}$	$V _{5}$	$V _{6}$	$V _{6}$
PL	N	$V _{3}$	$V _{4}$	$V _{4}$	$V _{5}$	$V _{5}$	$V _{6}$	$V_{ 6}$	$V_{ 1}$	$V_{ 1}$	$V _{2}$	$V _{2}$	$V_{ 3}$
NL		$V _{5}$	$V_{ 6}$	$V_{ 6}$	$V _{1}$	$V _{1}$	$V_{ 2}$	$V _{2}$	$V_{ 3}$	$V _{3}$	$V_{ 4}$	$V_{ 4}$	$V _{5}$
Z		$V _{7}$	$V _{0}$	$V _{7}$	$V _{0}$	$V _{7}$	$V _{0}$	$V _{7}$	$V _{0}$	$V_{ 7}$	$V_{ 0}$	$V_{ 7}$	$V _{0}$
PS		$V _{4}$	$V _{4}$	$V _{5}$	$V_{ 5}$	$V_{ 6}$	$V_{ 6}$	$V _{1}$	$V _{1}$	$V _{2}$	$V _{2}$	$V_{ 3}$	$V _{3}$
NS		$V_{ 5 }$	$V _{5}$	$V _{6}$	$V_{ 6}$	$V _{1}$	$V_{ 1}$	$V _{2}$	$V _{2}$	$V_{ 3}$	$V _{3}$	$V_{ 4}$	$V _{4}$

#### Inference

The inference method is performed using Mamdani's technique. The factor of weighting for i_th_ rule ($\alpha_{i}$) is given by:

18 $$\alpha_{i} = \min \left( {\mu Ai \left( {e\varphi } \right), \mu Bi\left( {eT} \right),\mu Ci\left( \theta \right)} \right)$$

19 $$\mu^{\prime}Vi\left( V \right) = \max \left( {\alpha i ,\mu Vi\left( V \right)} \right)$$

where $\mu Ai \left( {e\varphi } \right)$,$\mu Bi\left( {eT} \right) ,$ $\mu Ci\left( \theta \right)$ : flux, torque, and stator flux angle errors membership values.

#### Defuzzification

**Figure 6:**
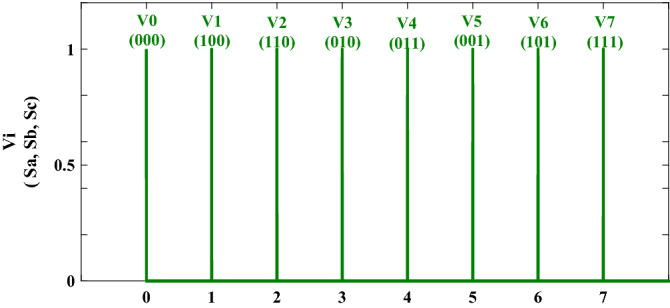
The membership functions for the output.

Figure 6 illustrates the obtained crisp values from the fuzzy values using the max method presented by Eq. (20).

20 $$\mu^{\prime}Vout\left( V \right) = max _{i = 1}^{180} \max \left( {\mu ^{\prime}Vi\left( V \right)} \right)$$

### Proposed loss minimization technique

**Figure 7:**
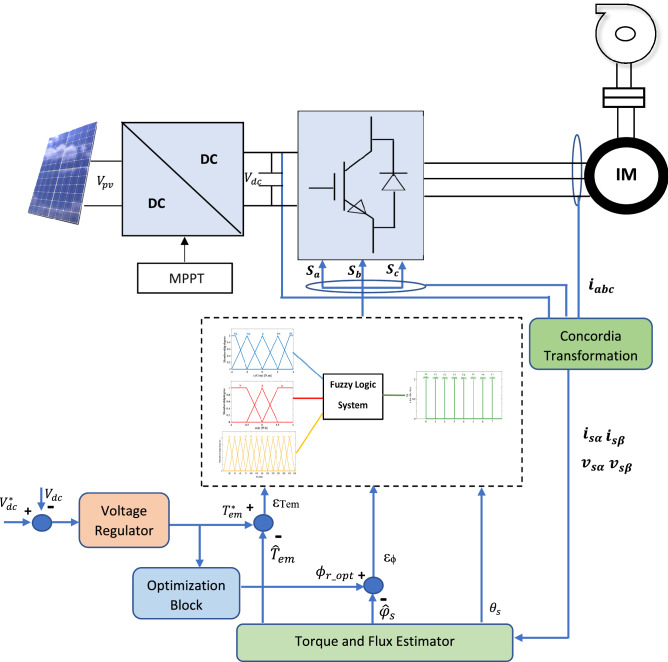
control scheme of PV water pumping system.

By improving the motor efficiency, it is possible to increase the flow rate and then the daily water pumped amount (Fig. 7). The purpose of the following technique is to associate a strategy based on losses minimization with the Direct Torque Control method.

It is well known that the value of the flux is important for the efficiency of the motor. A high value of the flux leads to an increase in the iron losses as well as a magnetic saturation circuit. On contrary, a low flux level leads to high joule losses.

Consequently, the reduction of the losses in the IM is directly linked with the choice of the flux level.

The proposed approach is based on the modeling of the joule losses in the machine which are related to the current flow through the stator windings. It consists in adjusting the value of the rotor flux to an optimal which minimizes the motor losses to increase efficiency. The joule losses can be expressed as follows (the core losses are neglected):

21 $$P_{j} = P_{j stator} + P_{j rotor}$$

22 $$P_{j stator} = R_{s} \left( {i_{ds}^{2} + i_{qs}^{2} } \right)$$

23 $$P_{j rotor} = R_{r} \left( {i_{dr}^{2} + i_{qr}^{2} } \right)$$

The total joule losses are given by:

24 $$P_{j} = R_{s} \left( {i_{ds}^{2} + i_{qs}^{2} } \right) + R_{r} \left( {i_{dr}^{2} + i_{qr}^{2} } \right)$$

A decrease in the current leads to a decrease in the joule losses. 

The electromagnetic torque $C_{em}$ and the rotor flux $\phi_{r}$ are calculated in d–q coordinate system as:

25 $$T_{em} = p \frac{M}{{L_{r} }}\phi_{r} i_{qs }$$

26 $$\phi_{r} = M i_{ds }$$

From Eqs. (25–26–27), the joule losses are as follows:

27 $$P_{joule} = R_{s} i_{ds}^{2} + \left( {(R_{r} \left( { \frac{M}{{L_{r} }}} \right)^{2} + R_{s} } \right)\left( { T_{em} \frac{{L_{r} }}{{Mp\phi_{r} { } }}} \right)^{2}$$

The electromagnetic torque $C_{em}$ and the rotor flux $\phi_{r}$ are calculated in reference (d,q) as:

28 $$T_{em} = p \frac{M}{{L_{r} }}\phi_{r} i_{qs }$$

29 $$\phi_{r} = M i_{ds }$$

By solving Eq. (30), we can find the optimal stator current which ensures both optimal rotor flux and minimal losses:

30 $$\frac{{d P_{joule} }}{{d i_{ds} }} = 0$$

Therefore, the optimal stator current is expressed by:

31 $$I_{dsopt} = K_{opt} i_{qs}^{*}$$

where

32 $$K_{opt} = \sqrt {1 + \left( {\frac{M}{{L_{r} }}} \right)^{2} \frac{{R_{r} }}{{R_{s} }}}$$

## Simulation results

Different simulations are conducted using MATLAB/Simulink software in order to evaluate the robustness and the performance of the proposed technique. The studied system is composed of eight CSUN 235-60P panels of 230 W (Table 2) connected in series. The centrifugal pump is driven by an IM which is characterized by the parameters presented in Table 3. The components of PV pumping system are listed in Table 4.

**Table 2:** Csun 235-60p PV panel characteristics.

Maximum power	235 W
Open circuit voltage	36.8 V
Short circuit current	8.59 A
Maximum power voltage	29.5 V
Maximum power current Pmax	7.97 A

**Table 3:** Induction machine characteristics.

$R_{s }$,$R_{r}$	4.85 (Ω), 3.805 (Ω)
$l_{s }$,$l_{r}$	0.274 [H], 0.274 [H]
Nominal power	1.5 (KW)
$P$	2
Inertia moment	0.031 (kg m^2^)
Viscous friction	0.00114 (N m s/rad)

**Table 4:** The used parameters of the PVWPS.

Parameter	Value
$V_{dc}^{*}$	400 V
$C_{dc}$	2000 μF
α	0.26
$L_{pv}$	3 mH

In this section, the PV water pumping system using FDTC with constant flux reference is compared with the proposed system based on the optimal flux (FDTCO) in the same operating conditions. The performances of both PV systems are tested by considering the following cases:

### Starting performance of the proposed system

**Figure 8:**
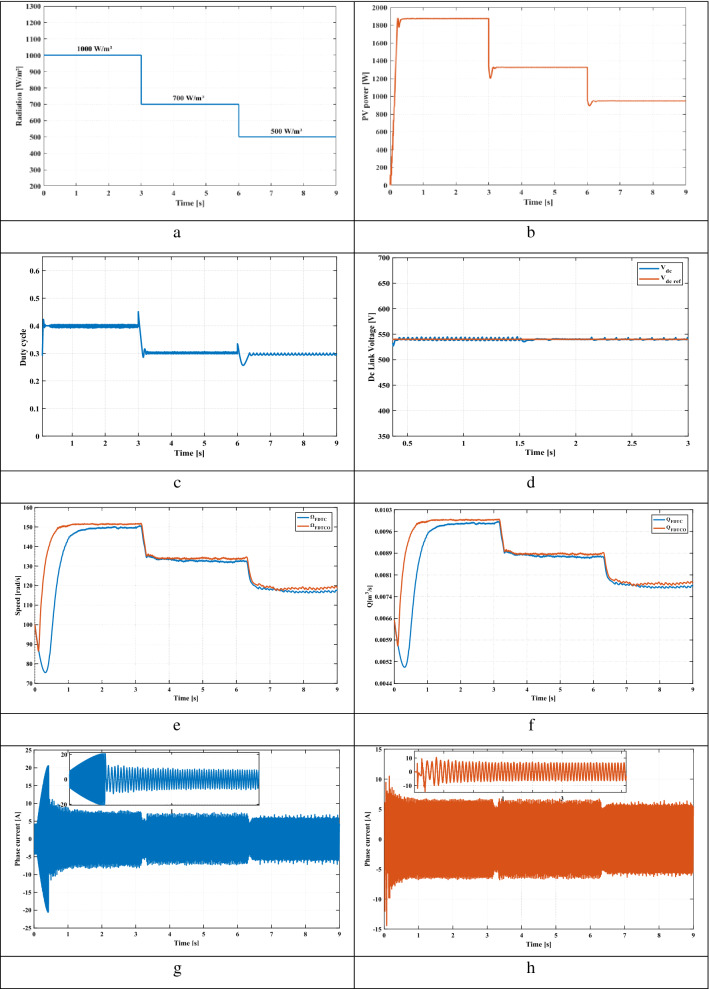

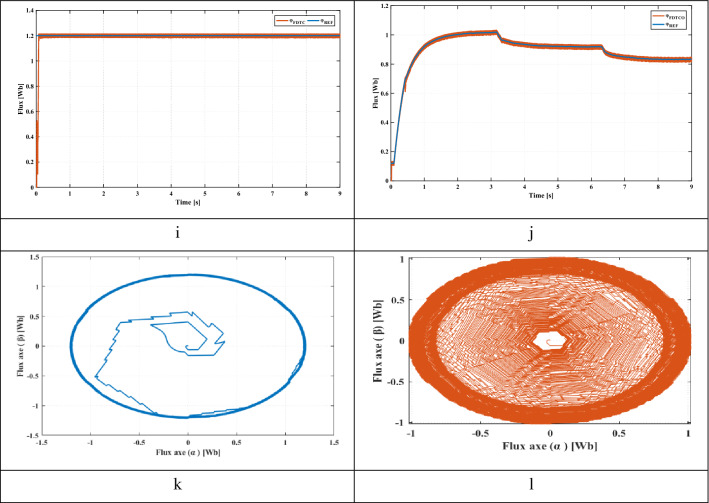
(**a**) Solar radiation (**b**) Extracted power (**c**) Duty cycle (**d**) DC link voltage (**e**) Rotor speed (**f**) Pumped water (**g**) Stator phase current of FDTC (**h**) Stator phase current of FDTCO (**i**) Flux response using FLC (**j**) Flux response using FDTCO (**k**) Stator flux trajectory using FDTC (**l**) Stator flux trajectory using FDTCO.

This section presents the starting up state of the proposed pumping system according to insolation of 1000 W/m^2^. Figure 8e illustrates the response of electric speed. The proposed technique provides a better rise time compared to FDTC, where the steady state is reached at 1.04 s while that with the FDTC, the steady state is reached at 1.93 s. Figure 8f shows the pumped water for both control strategies. It’s seen that, the FDTCO increases the pumped water, which explains the improvement of the energy converted by the IM. Figures 8g and 8h indicate the absorbed stator currents. The starting current is 20 A using the FDTC while the proposed control strategy indicates a starting current of 10 A, which leads in reducing the joule losses. Figures 8i and 8j show the developed stator flux. The PVPWS based on FDTC operates under a constant reference flux of 1.2 Wb while in the proposed method, the reference flux is 1A which involves improving the efficiency of PV systems.

### Variable solar irradiation

Solar radiation varies from 1000 to 700 W/m^2^ at 3 s, then to 500 W/m^2^ at 6 s (Fig. 8a). Figure 8b shows the PV power corresponding to 1000 W/m^2^, 700 W/m^2^ and 500 W/m^2^. Figures 8c and 8d illustrate the duty cycle and DC link voltage respectively. Figure 8e illustrates the electric speed of IM, we can notice that the proposed technique presents better speed and response time compared to the PV system based on FDTC. Figure 8f shows the volume of pumped water obtained using FDTC and FDTCO for various levels of irradiance. Using the FDTCO, it is possible to obtain a higher amount of pumped water than using FDTC. Figures 8g and 8h illustrate the simulated current response, with the FDTC method and the proposed control strategy. By using the suggested control technique, the current amplitude is minimized which implies the reduction of copper losses, which improves the system efficiency. Thus, a high stating current can cause a deterioration of the machine performance. Figure 8j presents the variation of developed flux responses in order to choose the optimum flux ensuring the minimization of the losses, thus, the suggested technique illustrates its performance. Contrary to Fig. 8i, the flux is constant which is not representing an optimal operation. Figures 8k and 8l indicate the evolution of the stator flux trajectory. Figure 8l illustrates the optimal flux development and explains the principal idea of the proposed control strategy.

### Sudden change in solar radiation

**Figure 9:**
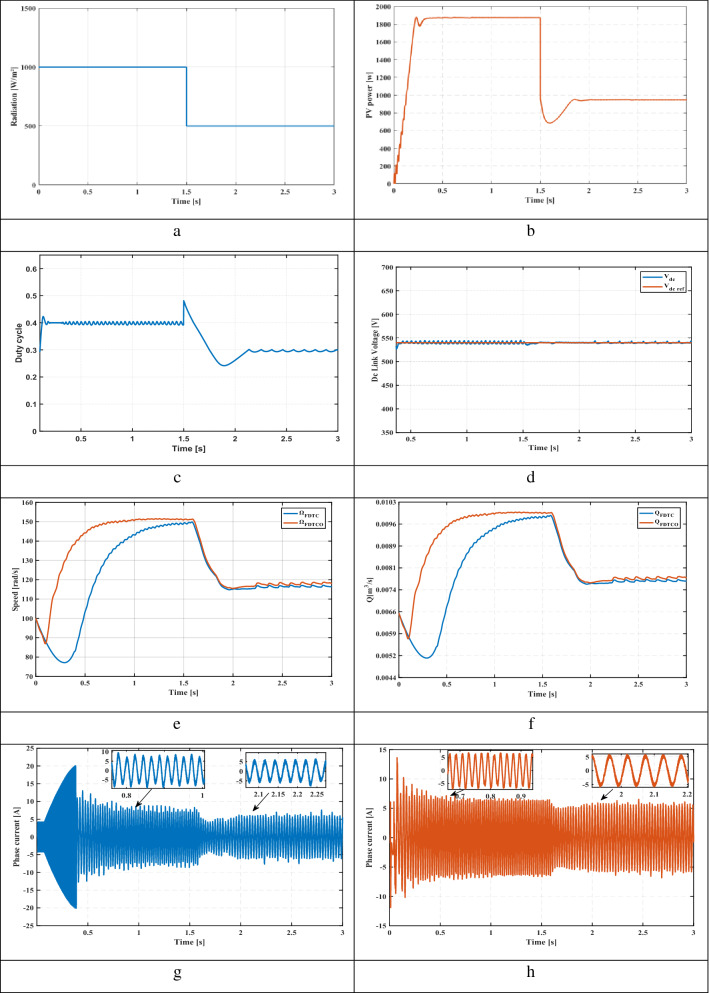

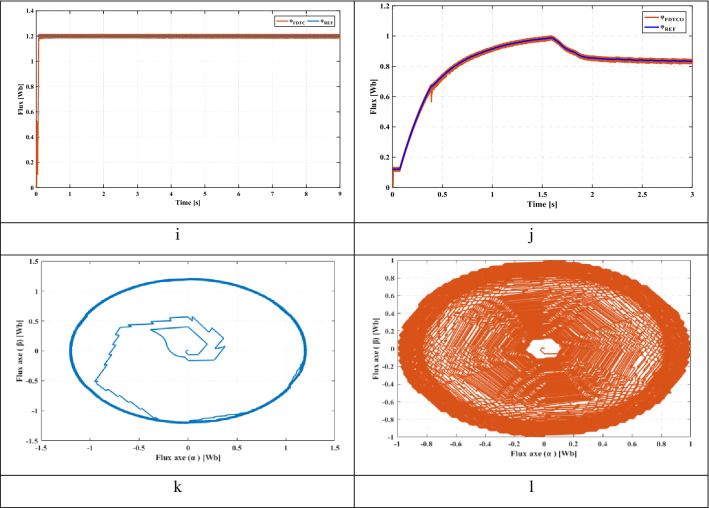
(**a**) Solar radiation (**b**) Extracted power (**c**) Duty cycle (**d**) DC link voltage (**e**) Rotor speed (**f**) water Flow (**g**) Stator phase current of FDTC (**h**) Stator phase current of FDTCO (**i**) Flux response using FLC (**j**) Flux response using FDTCO (**k**) Stator flux trajectory using FDTC (**l**) Stator flux trajectory using FDTCO.

A sudden change in solar radiation is applied, where the irradiance is 1000 W/m^2^ at the beginning, after 1.5 s, is decreased suddenly to 500 W/m^2^ (Fig. 9a). Figure 9b shows the extracted PV power from the PV panel corresponding to 1000 W/m^2^ and 500 W/m^2^. Figures 9c and 9d illustrate the duty cycle and DC link voltage respectively. From Fig. 9e, the proposed method offers a better response time. Figure 9f shows the pumped water volume obtained for both control strategies. Using the FDTCO, the amount of pumped water is higher than using FDTC, where the pumped volume water is 0.01 m3/s when the irradiance is 1000 W/m^2^ while, the pumped volume water is 0.009 m^3^/s for FDTC; moreover, the pumped volume water is 0.0079 m^3^/s for FDTCO when the irradiance is 500 W/m^2^, while the pumped volume water is 0.0077 m^3^/s for FDTC. Figures 9g and 9h. illustrate the simulated current response using the FDTC method and the suggested control strategy. We can notice that the proposed control strategy indicates a reduction in current amplitude under sudden irradiance variations, which leads in reducing the copper losses. Figure 9j presents the variation of developed flux responses in order to choose the optimum flux ensuring the minimization of the losses, thus, the proposed technique illustrates its performance, where the flux is 1Wb where the irradiance is 1000 W/m^2^ while, the flux is 0.83Wb where the irradiance is 500 W/m^2^. Contrary to Fig. 9i, the flux is constant of 1.2 Wb which does not represent an optimal functioning. Figures 9k and 9l indicate the evolution of the stator flux trajectory. Figure 9l illustrates the optimal flux development and explains the principal idea of the proposed control strategy and the improvement of the proposed water pumping system.

The comparative analysis of both techniques in terms of flux value, current amplitude and pumped water are presented in Table 5, which illustrates that PVWPS based on the proposed technique provides high performances with increasing pumped water flow, minimizing the amplitude current and losses, owing to the optimal flux choice.

**Table 5:** Comparative results between FDTC and FDTCO.

Irradiation (W/m^2^)	Performance					
	FDTC			FDTCO		
	Current (A)	Flux (Wb)	Water flow rate (m^3^/s)	Current (A)	Flux (Wb)	Water flow rate (m^3^/s)
1000	7.764	1.2	0.009	6.15	1	0.01
700	7.27	1.2	0.0087	5.94	0.9	0.0089
500	6.7	1.2	0.0077	5.8	0.83	0.0079

## PIL test using STM32F4 discovery board

**Figure 10:**
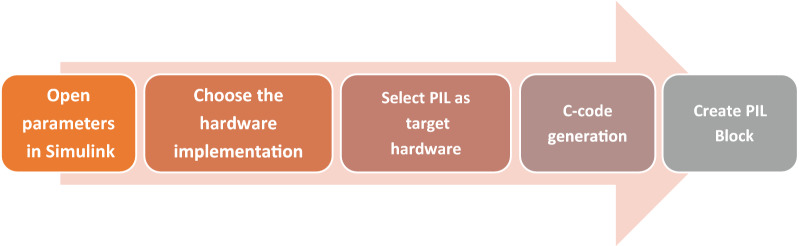
Steps to parameterize PIL test using STM32F407 MCU.

In order to validate and test the proposed control strategy, the PIL test based on the STM32F4 board is effectuated. It consists in generating the code that will be loaded and run on an embedded board. This board contains a 32-bit microcontroller with 1 Mbyte flash memory, clock frequency of 168 MHz, floating point unit, DSP instructions, 192 Kbytes SRAM. During this test, a developed PIL block is created in the control system incorporating the generated code based on the STM32F4 discovery hardware board and introduced on Simulink software. The steps allowing to configure the PIL test using STM32F4 board are illustrated in Fig. 10.

The co-simulation PIL test using STM32F4 can be utilized as a low-cost technique to validate the proposed technique. In this paper, the optimization block which provides the optimal reference flux is executed in the STMicroelectronics Discovery board (STM32F4).

This latter and Simulink and are executed at the similar period and exchanged the information using of proposed method for PVWPS in the co-simulation process. Figure 12 illustrates the implementation of the optimization technique subsystem in STM32F4.

Only the proposed technique of the optimal reference flux has been displayed in this co-simulation because it is the main control variable of this work that demonstrates the PV water pumping system control behavior.

**Figure 11:**
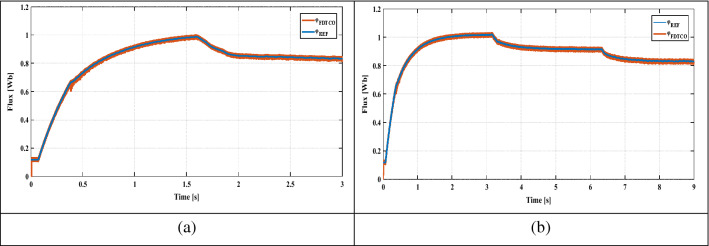
PIL test results for flux response.

**Figure 12:**
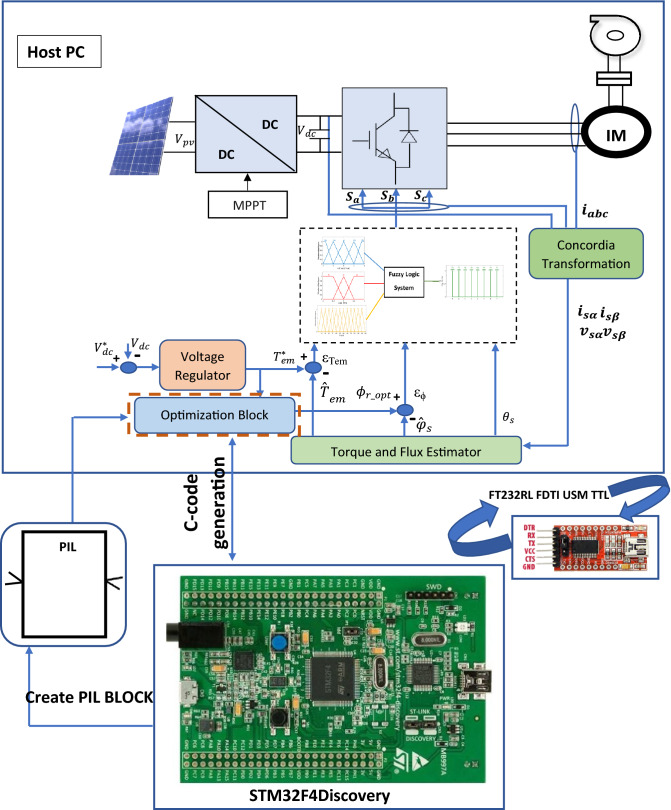
PIL test of the optimal reference flux block using STM32F4 board.

Figure 11a,b show the PIL test results for the proposed method under Variable and sudden change in solar radiation. The numerical simulation results indicate a similar behavior to those obtained through PIL co-simulation test, showing that the proposed control strategy is powerful (Fig. 12). Consequently, the co-simulation PIL process can be utilized as an experimental setup to validate the hardware implementation of various control strategies.

## Conclusion

An improved DTC strategy for PVPWS applications is presented here. The proposed technique is based on FLC which aims to operate the motor at the optimal value of flux. The simulation has been carried out in Matlab/Simulink to evaluate the performance of the proposed control strategy and compared to FDTC with constant flux reference under different operating conditions. A co-simulation PIL validation based on STM32F4 board has been carried out. Numerical simulation results indicate a similar behavior to those obtained through the PIL co-simulation test, which can be employed as a great implement, experimental setup and low-cost technique to evaluate the control strategies.

According to the obtained results, the main improvements are:

- The stator current is reduced, consequently the motor losses are minimized.
- The optimizing of the rotor flux
- The pumped water is increased under different operating conditions
- The efficiency of the proposed PV water pumping system is improved.
